# Evaluation of the Demineralization Development around Different Types of Orthodontic Brackets

**DOI:** 10.3390/ma16030984

**Published:** 2023-01-20

**Authors:** Melis Toz Ertop, Orhan Cicek, Hande Erener, Nurhat Ozkalayci, Busra Demir Cicek, Fusun Comert

**Affiliations:** 1 Department of Orthodontics, Faculty of Dentistry, Zonguldak Bulent Ecevit University, 67100 Zonguldak, Turkey; 2 Department of Orthodontics, Faculty of Dentistry, Tekirdag Namık Kemal University, 59030 Tekirdağ, Turkey; 3 Department of Healthcare Management, Boyabat Faculty of Economics and Administrative Sciences, Sinop University, 57000 Sinop, Turkey; 4 Department of Endodontics, Faculty of Dentistry, Zonguldak Bulent Ecevit University, 67100 Zonguldak, Turkey; 5 Department of Medical Microbiology, Faculty of Medicine, Zonguldak Bulent Ecevit University, 67630 Zonguldak, Turkey

**Keywords:** orthodontics, bracket, bond, artificial saliva, *Streptococcus mutans*, cariogenic environment, demineralization, DIAGNOdent, dentistry

## Abstract

The aim of this study was to compare the demineralizations of the enamel surfaces around different types of orthodontic brackets in an artificial cariogenic environment. A total of 90 extracted human maxillary first premolar teeth were used in this in vitro study. The teeth were divided into 6 groups, 5 study and 1 control, each consisting of 15 samples. Victory metal, Gemini metal, Clarity self-ligating ceramic, APC Clarity Advanced ceramic and Clarity Advanced ceramic brackets (3M Unitek, Monrovia, Calif) used in the study groups were bonded to the teeth with the direct technique. The gingival, occlusal and proximal enamel surfaces adjacent to the brackets were measured with a DIAGNOdent pen (KaVo, Biberach, Germany) (T0). Then, the teeth were placed in a cariogenic suspension environment containing *Streptococcus mutans*, sucrose and artificial saliva. The teeth were removed from the cariogenic suspension at the end of 28 days. Enamel surfaces were remeasured with DIAGNOdent and the values were recorded (T1). Whether the obtained data were homogeneously distributed or not was determined by the Kolmogorov–Smirnov test, within-group comparisons were performed with the Wilcoxon test, and between-group comparisons were performed with Mann–Whitney U and Kruskal–Wallis tests. Significance level was accepted as *p* < 0.05. In all groups, the demineralization values of the enamel surfaces in the gingival, proximal and occlusal surfaces adjacent to the brackets were significantly higher in the T1 period than in the T0 period (*p* < 0.05). In the T1 period of Gemini metal, Clarity self-ligating ceramic and Clarity advanced ceramic bracket groups, the demineralization values of the proximal enamel surfaces were found to be significantly higher than the Victory metal and APC Clarity Advanced ceramic bracket groups (*p* < 0.05). In the T1 period, the demineralization values of the occlusal enamel surfaces of the Victory metal, APC Clarity Advanced ceramic bracket groups and control group were significantly lower than the Gemini metal, Clarity self-ligating ceramic and Clarity Advanced ceramic bracket groups (*p* < 0.05). Significant increases in enamel demineralization values were observed as a consequence of increased retention areas for microbial dental plaque on enamel surfaces adjacent to the bracket. Considering the importance of minimizing enamel demineralization in fixed orthodontic treatments, less enamel demineralization in Victory metal and APC Clarity Advanced ceramic bracket groups showed that these brackets can be preferred in patients with poor oral hygiene.

## 1. Introduction

Orthodontic treatment aims to correct the positions of the teeth according to the jaws, jaws to each other and to the base of the cranium, and thus to ensure the ideal aesthetics and function of the teeth [1]. With the increase in the importance given to aesthetic appearance in society, applications to dentists with aesthetic demand have increased and orthodontic treatments have become more common [2].

Orthodontic treatment includes the use of both fixed and removable appliances separately or together [2]. Brackets are the most important element responsible for transmitting the force to the teeth in fixed orthodontic treatment. Brackets for fixed orthodontic treatment can be classified according to (i) material (stainless steel, titanium or ceramic, plastic); (ii) production technique (drawing, casting, sintering, metal injection molding); (iii) size (mini, ultra mini etc.); (iv) base shapes (straight, curved); (v) widths (narrow, wide); (vi) shapes (vertical slotted, double slotted or according to the technique they are applied like begg, tip edge); (vii) ligating methods with archwire (self-ligating and conventional) [3,4].

In fixed orthodontic treatment, bands and brackets bonded to the teeth increase the adhesion of plaque and food to smooth tooth surfaces, which tend to have a low prevalence of caries [5]. The irregular surfaces of brackets, bands and wires also limit the natural cleaning mechanism of oral muscles and saliva [6]. In addition, these orthodontic attachments complicate the mechanical removal of bacterial plaque by the patient [5,7].

For the formation of tooth caries, cariogenic bacteria, a vulnerable tooth surface and nutrients to support bacterial growth must be present at the same time [8]. *Streptococcus mutans*, one of more than 300 bacterial species found in the oral cavity, is a cariogenic organism that causes caries. Cariogenic bacteria are major factors of the initial caries; they adhere to enamel, produce and tolerate acid, proliferate and develop in a sucrose-rich environment [8].

It is seen that various methods are used to create artificial caries in studies conducted to examine enamel demineralization and white spot lesions associated with fixed orthodontic appliances. Existing caries simulation models can be classified as in vitro demineralization using acid buffers, in vitro demineralization using acids produced by bacteria, in vitro demineralization/remineralization created using a pH cycle, an artificial mouth in which the acid threat, produced by bacteria, is diluted with artificial saliva solution, in vivo animal studies, in situ demineralization/remineralization using blocks or sections of enamel or dentin in the human mouth and in vivo studies using teeth planned to be extracted in the human mouth [9]. The solid surface layer and subsurface lesion characteristics of initial enamel lesions cannot be imitated with in vitro demineralization solutions. With these solutions, erosion-type deterioration occurs on the enamel surface. The use of a cariogenic environment with bacterial culture and artificial saliva components may produce caries similar to the initial enamel lesion characteristic [10].

As a result of the inability to remove bacterial plaque from retentive tooth areas, a diet containing abundant refined carbohydrates and frequent carbohydrate intake, the dynamic balance between demineralization and remineralization in tooth enamel is disrupted in favor of demineralization and clinically detectable white spot lesions develop [11]. Changes in light scattering in decalcified and porous enamel cause a white appearance [12]. White spot lesions are a common side effect of orthodontic treatment with fixed appliances and can usually occur within four weeks, the time interval between two orthodontic treatment appointments [5,7].

Early diagnosis of white spot lesions is very important so that they can be detected before tooth integrity is deteriorated and appropriate preventive treatment procedures can be applied. It is thought that the combination of conventional methods used in routine applications and recently developed current methods will facilitate the early diagnosis of white spot lesions. Examination with mirror and probe, visual inspection and radiographic examination are conventional caries detection methods used in routine. Digital radiography, electrical caries monitoring (ECM), fiber optic transillumination, ultrasonic caries detector, alternating current impedance spectroscopy, laser fluorescence, quantitative light-induced fluorescence (QLF) and reflectance confocal microscopy (RCM) are among the current methods [13,14].

The aim of this study was to compare the enamel demineralizations developing around different types of orthodontic brackets bonded to the extracted human maxillary first premolars before they were placed in the artificial cariogenic suspension environment and 28 days after. We tried to imitate the oral environment as much as possible, and the ambient conditions were equalized for all samples. WSL can occur within 4 weeks, which is usually the time between two orthodontic treatment appointments [5,7]. For this reason, the duration of the experiment was determined as 28 days. The use of lasers is utilized in many areas of dentistry, including the treatment of dentine hypersensitivity [15]. DIAGNOdent pen, a laser fluorescence method, was used to detect demineralizations in the study.

## 2. Materials and Methods

### 2.1. Ethical Approval and Preparation of Samples

**Figure 1:**
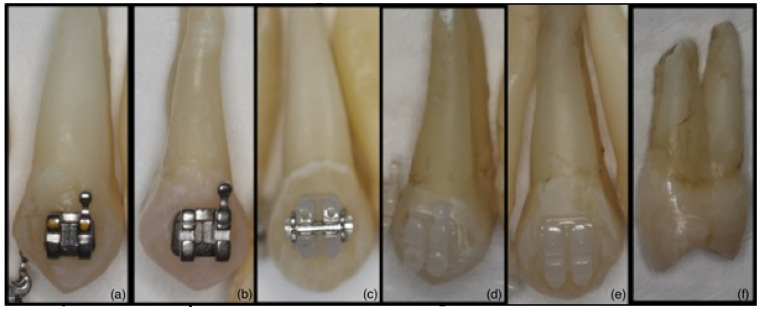
(**a**): Victory metal (Group 1); (**b**): Gemini metal (Group 2); (**c**): Clarity self-ligating ceramic (Group 3); (**d**): APC Clarity Advanced ceramic (Group 4); (**e**): Clarity Advanced ceramic (Group 5); (**f**): Control (Group 6).

Ethics committee approval for the study was given by the Non-Invasive Clinical Research Ethics Committee at Zonguldak Bulent Ecevit University (18/11/2020:2020/22). The sample size of the study, in which the α error probability was set as 0.05, the power was 0.95, and the effect size was calculated using the mean and standard deviation of the groups, was performed with G*Power 3.1.9.7 program. Based on these data, the actual power of the study was calculated as 97% and the total sample size should be 42. The primary endpoint of the study was that the demineralizations in the samples did not develop in the cariogenic suspension environment. The study was performed using 90 maxillary premolars extracted for orthodontic treatment from patients with good oral hygiene. The teeth included in the study were free of caries, fillings, restorations, cracks, fractures, dental extraction forceps marks and fluorosis on the enamel [16]. The teeth were stored in glass bottles containing 0.1% thymol solution at room temperature, in a dark and closed environment, for a maximum of 6 months until the study time [16]. The teeth removed from thymol solution were divided into 6 groups consisting of 15 samples in each group. In this study, representation of groups is given in Figure 1, respectively: In Group 1, Victory metal (Figure 1a); in Group 2, Gemini metal (Figure 1b); in Group 3, Clarity self-ligating ceramic (Figure 1c); in Group 4, Adhesive Precoated (APC) Clarity Advanced ceramic (Figure 1d); and in Group 5, Clarity Advanced ceramic brackets (Figure 1e) (3M Unitek, Monrovia, Calif) were bonded to the teeth by direct bonding (etch-and-rinse) technique. Group 6 was considered as the control group without brackets (Figure 1f).

Soft residues on all surfaces of teeth were removed before the brackets were bonded, and buccal and palatal surfaces were cleaned using a slow speed air-cooled micromotor (KaVo Dental GmbH, Biberach/Riss, Germany) with pumice. The area where the bracket will be placed on the buccal enamel surfaces was closed with a 4 × 4 mm windowed acetate sheet. The limited enamel surface was etched and bonded with the use of acetate sheet. Thus, the retentive enamel surface area that may occur due to acid etching was minimized. In order to roughen the enamel surfaces, 37% phosphoric acid in blue color (Panora 200, Imicryl, Turkey) was applied to the enamel surface, which is the mesio-distal and inciso-gingival center of the clinical crown, where the bracket will be placed, for 30 s. Then, washing for 15 s and drying for 10 s were performed [17]. After the white chalky surface was seen, Transbond XT Light Cure Adhesive Primer (3M Unitek, Monrovia, CA, USA) was applied to the tooth surfaces as a thin layer. APC brackets were removed from their boxes and attached to the teeth. Light-cured Transbond XT Light Cure Adhesive Paste (3M Unitek, Monrovia, CA, USA) was used for bonding all brackets except APC brackets. Brackets loaded with adhesive paste on their base were carefully placed on all tooth surfaces on which adhesive primer was applied. Flashes were gently removed with a thin probe. 3M Espe Elipar S10 (3M ESPE Dental Products) light source with a light intensity of 1200 Mw/cm^2^ and a wavelength of 430–480 nm was used for polymerization. Polymerization was achieved by applying light to the brackets for a total of 20 s, 10 s from the mesial and distal.

### 2.2. Measurement of Samples with DIAGNOdent before Placing in Cariogenic Suspension Environment (T0)

**Figure 2:**
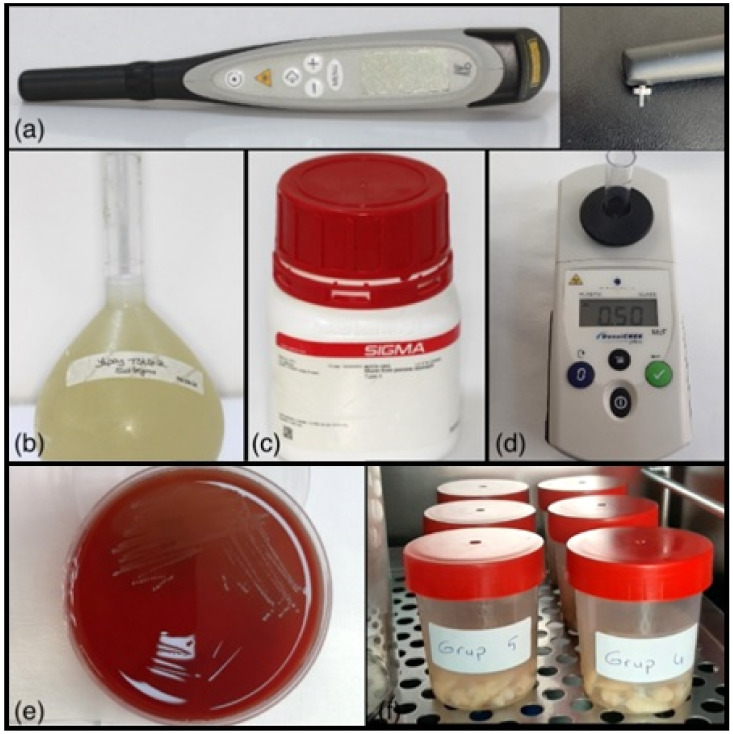
(**a**): DIAGNOdent pen and tip used in the study (KaVo, Biberach, Germany); (**b**): Artificial saliva solution; (**c**): Mucin, Type II; Sigma-Aldrich; (**d**): DensiCHEK plus (device for measuring the optical density suspension of microorganisms); (**e**): Streptococcus mutans culture; (**f**): Sample tubes placed in the incubator.

Prior to applying DIAGNOdent pen measurement procedure (KaVo, Biberach, Germany), it was calibrated according to the manufacturer’s instructions. After pressing the calibration button, when the signal is heard, the tip is placed vertically on the calibration plane. The calibration is completed when the signal stops. Measurements were performed by the same researcher (M.T.E.) in order to avoid errors due to measurements by different people. In addition, the measurements were repeated twice. The DIAGNOdent pen device detects its readings as fluorescence a.u. (arbitrary units) and creates a score. Demineralization values on the gingival, mesial, distal and occlusal enamel surfaces around the brackets in the study groups and on the enamel surfaces, where the brackets were estimated to be placed in the control group, were measured using the DIAGNOdent pen. Measurements were performed with the cylindrical 1.1 mm diameter tip no. 2 of the DIAGNOdent pen device designed for smooth surfaces [18] (Figure 2a). After the tip of the DIAGNOdent pen was moved back and forth in the gingival and occlusal surfaces, up and down in the mesial and distal surfaces, the highest value seen on the LED screen was recorded as T0 for each group separately [19,20]. The values obtained from the mesial and distal surfaces were averaged and evaluated as a single proximal value [21].

### 2.3. Preparation of Artificial Saliva

Artificial saliva was prepared as 0.4 g sodium chloride (NaCl), 0.4 g potassium chloride (KCl), 0.8 g calcium chloride (CaCl_2_·2H_2_O), 0.78 g sodium di hydrogen phosphate (NaH_2_PO_4_·2H_2_O), 0.005 g sodium sulphate (NaS·9H_2_O) and 1 g urea in 1000 mL deionized water (g/L) (Figure 2b), [22]. After the prepared artificial saliva was sterilized in an autoclave, 140 mg of mucin (Mucin from pork stomach, Type II; Sigma-Aldrich Chemie GmbH, Deisenhofen, Germany) (Figure 2c) was added to 100 mL of artificial saliva. It was aimed to accelerate the pellicle formation on the samples with the addition of mucin [23].

### 2.4. Preparation of Artificial Cariogenic Suspension Environment

*Streptococcus mutans* culture was used to from the cariogenic suspension (Figure 2e). The bacterial culture used in the present study was prepared in accordance with the bacterial suspension and broth used in an in vitro study by Hayati et al. [24] to create artificial biofilm-induced caries. Bacteria resuscitated from stock culture were grown in brain–heart infusion broth containing 1% glucose by incubating at 37 °C for 18 h under 10% CO_2_ atmospheric conditions. With the bacteria taken from here, a bacterial suspension was formed in the brain–heart infusion broth, equivalent to 0.5 McFarland (10^8^ cfu/mL) turbidity (Figure 2d), for each tube. Bacterial suspension was prepared in artificial saliva and sucrose solution (1 g sucrose/10 mL distilled water) at 10^6^ cfu/mL turbidity [25]. Teeth were prepared in such a way that each group was in a separate tube. The teeth were placed in a cariogenic suspension containing 49 mL of artificial saliva, 0.5 mL of bacterial suspension and 0.5 mL of sucrose in each tube and incubated in an incubator for 28 days at 37 °C under 10% CO_2_ atmosphere [5,7,21,22] (Figure 2f). The cariogenic suspension environment of the samples was renewed every 48 h [10].

### 2.5. Measurement of Samples with DIAGNOdent 28 Days after Placement in Cariogenic Suspension Environment (T1)

At the end of 28 days, the demineralizations on the enamel surfaces were measured with a DIAGNOdent pen in the same way as in T0, and recorded as T1.

### 2.6. Statistical Analyses

SPSS 27.0 (SPSS, Inc., Chicago, IL, USA) program was used for statistical analysis of the data obtained in the study. Mean, standard deviation, median, minimum, maximum value, frequency and percentage were used for descriptive statistics. Whether the data was homogeneously distributed or not was evaluated with the Kolmogorov–Smirnov test. Wilcoxon test was used for intragroup comparison; Mann–Whitney U and Kruskal–Wallis tests were used for intergroup comparisons. Mann–Whitney U test was used for non-normal distributions. Significance level was accepted as *p* < 0.05.

## 3. Results

The statistical comparisons of the demineralization values of the gingival, proximal and occlusal enamel surfaces adjacent to the bracket at the T0 and T1 periods of the samples are given in Table 1, Table 2, Table 3 and Table 4. The results show that a significant increase was found in the demineralization values on the gingival, proximal and occlusal enamel surfaces adjacent to the bracket in all groups in the T1 period compared to the T0 period (*p* < 0.05). In the T1 period of the control group, demineralization values on the gingival and proximal enamel surface adjacent to the bracket were found to be significantly lower than the study groups (*p* < 0.05) (Table 1 and Table 2). From the T0 period to the T1 period, the amount of increase in demineralization values occurring on the gingival and proximal enamel surface adjacent to the bracket of the study groups was found to be significantly higher than the control group (*p* < 0.05) (Table 1 and Table 2). In the T1 period, the demineralization values on the occlusal enamel surface adjacent to the bracket of the Gemini metal, Clarity self-ligating ceramic and Clarity Advanced ceramic bracket groups were found to be significantly higher than the control group (*p* < 0.05) (Table 3). From the T0 to T1 period, the amount of increase in demineralization values was found to be significantly lower in the control group than those in the Victory metal, Gemini metal, Clarity self-ligating ceramic and Clarity Advanced ceramic bracket groups (*p* < 0.05) (Table 3 and Table 4).

### 3.1. Comparison Results by Bracket Type

#### 3.1.1. Comparison of Metal Brackets

The demineralization values of the gingival and occlusal enamel surface adjacent to the bracket in the T1 period of the Victory metal bracket group were found to be significantly lower than the Gemini metal bracket group (*p* < 0.05) (Table 1 and Table 3). The demineralization values of the proximal enamel surface adjacent to the bracket in the T1 period of the Victory metal bracket group were found to be lower than the Gemini metal bracket group. However, the difference was not statistically significant (*p* > 0.05) (Table 2).

As seen in Table 1, Table 2 and Table 3, there was no significant difference between the amount of increase in demineralization values occurring on the adjacent gingival, proximal and occlusal enamel surfaces of the Victory metal and Gemini metal bracket groups from the T0 period to the T1 period (*p* > 0.05).

#### 3.1.2. Comparison of Ceramic Brackets

In the T1 period, the demineralization values of the APC Clarity Advanced ceramic bracket group were found to be significantly lower than the Clarity Advanced ceramic and Clarity self-ligating ceramic bracket groups (*p* < 0.05) (Table 1, Table 2 and Table 3). From the T0 to T1 period, the amount of increase in demineralization values was found to be significantly higher than Clarity self-ligating ceramic and APC Clarity Advanced ceramic bracket groups (*p* < 0.05), (Table 1). In the similar range of the T0 to T1 period, the amount of increase in demineralization values was found to be significantly higher than the APC Clarity Advanced ceramic bracket group (*p* < 0.05) (Table 2 and Table 3). From the T0 period to the T1 period, the amount of increase in demineralization values occurring on the proximal enamel surface of the Clarity self-ligating ceramic bracket group was found to be significantly lower than in the Clarity Advanced ceramic bracket group (*p* < 0.05) (Table 2).

#### 3.1.3. Comparison of Metal and Ceramic Brackets

In the T1 period, the demineralization values of the Victory metal bracket group on the gingival enamel surface adjacent to the bracket were found to be significantly lower than the APC Clarity Advanced ceramic, Clarity Advanced ceramic and Clarity self-ligating ceramic bracket groups (*p* < 0.05) (Table 1). In the T1 period, the demineralization values of the Gemini metal bracket group on the gingival enamel surface adjacent to the bracket were found to be significantly lower than the Clarity Advanced ceramic and Clarity self-ligating ceramic bracket groups (*p* < 0.05) (Table 1). From the T0 period to the T1 period, it was found to be significantly higher than the metal bracket groups (*p* < 0.05) (Table 1 and Table 2). In the T1 period of the Victory metal bracket group, the demineralization values on the proximal and occlusal enamel surfaces adjacent to the bracket were found to be significantly lower than the Clarity Advanced ceramic and Clarity self-ligating ceramic bracket groups (*p* < 0.05). From the T0 period to the T1 period, the amount of increase in demineralization values was found to be significantly lower than the other study groups (*p* < 0.05) (Table 2 and Table 3).

### 3.2. Comparison of Brackets by Ligating Type (Conventional and Self-Ligating)

In the T1 period, the demineralization values of the Clarity self-ligating ceramic bracket group on the gingival enamel surface adjacent to the bracket were found to be significantly higher than all conventional bracket groups, except for Clarity Advanced ceramic (*p* < 0.05) (Table 1). From the T0 to T1 period, the amount of increase in the demineralization values occurring was found to be significantly higher than the Victory metal group, and significantly lower than the Clarity Advanced ceramic bracket group (*p* < 0.05) (Table 1).

The demineralization values of the Clarity self-ligating ceramic bracket group in the T1 period were found to be significantly higher than the Victory metal and APC Clarity Advanced ceramic bracket groups (*p* < 0.05) (Table 2 and Table 3). From the T0 period to the T1 period, the amount of increase in demineralization of the Clarity self-ligating ceramic bracket group was found to be significantly higher than the APC Clarity Advanced ceramic bracket group (*p* < 0.05) (Table 2 and Table 3). From the T0 period to the T1 period, the amount of increase in demineralization values of the Clarity self-ligating ceramic group was found to be significantly lower than the Clarity Advanced ceramic bracket group (*p* < 0.05) (Table 2).

## 4. Discussion

Initial caries lesions are called white spot lesion (WSL). The formation of WSL occurs when the dynamic balance between demineralization and remineralization is disrupted in favor of demineralization as a result of the inability to remove plaque from the retentive tooth areas, a diet containing abundant refined carbohydrates and frequent carbohydrate intake [5,11]. Bands and brackets placed on the teeth in fixed orthodontic treatment increase the retention of plaque and food to smooth tooth surfaces, which tend to have a low prevalence of caries [5]. WSL can occur within 4 weeks, which is usually the time between two orthodontic treatment appointments [5,7]. In the present study, a significant increase was found in the demineralization values of the gingival, proximal and occlusal enamel surfaces adjacent to the bracket 28 days after the samples were placed in the cariogenic environment in all groups.

Chatterjee and Kleinberg [26] concluded that pH, calcium and phosphate levels decreased and carbohydrate levels increased in plaque after the placement of orthodontic appliances, regardless of the area examined in the mouth of patients undergoing orthodontic treatment. In the in vitro study of Clarkson et al. [10] using oral bacteria to create caries-like lesions on enamel and dentin, it was observed that the pH was 5.2 when the teeth were first placed, and 4.55 for all tubes after 48 h of incubation. In the presented study, 48 h after the samples were placed in the cariogenic suspension environment, a decrease in the pH of the cariogenic suspension was observed. The pH value of the newly prepared cariogenic suspension was found to be 7.2. The pH value of the 48 h suspension collected for replacement was found to be 5.1.

There are many studies in the literature investigating the relationship between fixed orthodontic treatment and white spot lesion [6,27,28,29,30,31,32]. In the study of Hadlerolsen et al. [6], it was concluded that the risk of developing white spot lesion is higher in people with orthodontic treatment compared to untreated people. Tufekci et al. [33], in their study, compared patients who received orthodontic treatment for 6, 12 months and the control group in terms of white spot lesion formation. In the 6- and 12-month treatment groups, the percentages of individuals with at least one visible white spot lesion were 38% and 46%, respectively. They reported that only 11% of the individuals in the control group had at least one white spot lesion. Lucchese and Gherlone [27], in their study, reported that there was significantly decalcification in pediatric patients 6 months after the bonding of fixed orthodontic attachments. Akın et al. [29], in their study to investigate the incidence of white spot lesions during fixed orthodontic treatment, reported that the prevalence of white spot lesions was 21% before fixed orthodontic treatment, and that white spot lesions were seen in 65% of patients after fixed orthodontic treatment. In a cross-sectional study to determine the prevalence and severity of enamel opacities in patients before and after orthodontic treatment, Mizrahi [30] concluded that there was a significantly increase in both prevalence and severity after completion of orthodontic treatment. In the present study, the demineralization values measured in the T1 period in the estimated gingival and proximal enamel surfaces adjacent to the bracket in the control group without bracket were found to be significantly lower than in the study groups with brackets, consistent with the literature. Similarly, from the T0 period to the T1 period, the amount of increase in demineralization values occurring on the gingival and proximal enamel surface of the control group was found to be significantly lower than the study groups.

In the literature, there are studies examining the relationship between the amount of microbial plaque, the resulting white spot lesion and different types of materials used in fixed orthodontic treatment. According to the study of Eliades et al. [32], it was concluded that the microorganism binding potential on metal brackets is higher than on ceramic brackets. In the study of Almosa et al. [34], to compare the demineralization degrees of teeth bonded with metal and ceramic brackets, it was reported that teeth bonded with ceramic brackets showed significantly higher enamel demineralization compared to teeth bonded with metal brackets. In the study of Ahn et al. [35], to analyze the adhesion amount of streptococcal strain in different orthodontic brackets, it was reported that the adhesion amounts in stainless steel brackets were significantly higher than in ceramic brackets. In the clinical study of Lindel et al. [36], to evaluate whether there is a difference in biofilm adhesion between metal and ceramic brackets, it was reported that there is less biofilm in ceramic brackets than metal brackets. In the present study, from the T0 period to the T1 period, the amount of increase in demineralization values was found to be significantly higher in the Clarity Advanced ceramic bracket group than the metal bracket groups, consistent with the result of the study of Almosa et al. [34]. In our study, demineralization values of the gingival enamel surface adjacent to the bracket of the APC Clarity Advanced and Clarity self-ligating ceramic bracket groups were found to be significantly higher than the metal bracket groups in the T1 period. Moreover, the demineralization values of the gingival enamel surface adjacent to the bracket of the APC Clarity Advanced ceramic bracket group were found to be significantly higher than the Victory metal bracket group. On one hand, from the T0 period to the T1 period, the amount of increase in demineralization values occurring on the gingival enamel surface adjacent to the bracket of the Victory metal bracket group was found to be significantly lower than the ceramic bracket groups. On the other hand, from the T0 to T1 period, the amount of increase in demineralization values occurring on the gingival and proximal enamel surface adjacent to the bracket of the Clarity Advanced ceramic bracket group was found to be significantly higher than the metal groups. These results were similar to the results of the study by Almosa et al. [34]. In addition, from the T0 period to the T1 period, the amount of increase in demineralization values of the APC Clarity Advanced ceramic bracket group was found to be significantly lower than the metal bracket groups, which is consistent with the result of Eliades et al.’s [32] study.

It is common for a certain amount of adhesive flash to remain between the bracket and the enamel while the brackets are bonded. The effect of this situation on the formation of white spot lesions has been investigated in various studies [37]. It has been shown that bacteria will readily colonize the surface of rough materials such as composites, potentially increasing the incidence of white spot lesions [38]. It is important to remove adhesive flash so that it can reduce the plaque accumulation and the incidence of the white spot lesions. Armstrong et al. [37], in their study comparing APC PLUS brackets and conventional brackets, reported that there was no significant difference between the two groups in terms of residual adhesive amount. Guzman et al. [39] compared APC II brackets with conventional brackets in terms of residual adhesive and observed that less adhesive remained in APC II brackets after debonding. Tan and Çokakoglu [21], in their study to evaluate the effects of APC Flash-free brackets on enamel demineralization, reported that there was no difference between the effects of APC Flash-free and conventional ceramic brackets on enamel demineralization. In the present study, on all enamel surfaces adjacent to the bracket, increasing demineralization values were found to be significantly lower in the APC Clarity Advanced ceramic bracket group than in the Clarity Advanced ceramic bracket group.

Recent research showed that Biomimetic Hydroxyapatite compounds showed deposition of hydroxyapatite on polymeric composite, thus preventing caries on the margins of composite frameworks such as at the bracket/enamel interface [40]. In a different study, Casein phosphopeptide-amorphous calcium phosphate was found to be effective in remineralizing early enamel caries at the surface level [41]. Future similar laboratory and clinical studies are needed in order to test also the efficacy of these compounds.

In a clinical study comparing self-ligating and conventional brackets in terms of plaque accumulation, it was concluded that there was no significant difference between the visual plaque index of the two bracket groups [42]. Similarly, in the present study, no significant difference was found between the demineralization values of the Clarity Advanced ceramic and Clarity self-ligating ceramic bracket groups in the T1 period. There was no significant difference between the amount of increase in the demineralization values occurring on the occlusal enamel surfaces adjacent to the bracket of the Clarity self-ligating ceramic and Clarity Advanced ceramic bracket groups, from the T0 period to the T1 period.

The limitations of this study are the use of brackets from a single manufacturer, the use of a single bonding technique and the inadequacy of the artificial cariogenic environment to fully simulate the oral flora. However, in our study, erosion-type deteriorations on the enamel surface created by in vitro demineralization solutions did not occur, and the intact surface layer and sub-surface lesion characteristics of the initial enamel lesions could be simulated [43]. In order to eliminate the inadequacy of clinical trials about this topic, it would be beneficial to conduct further studies by considering the limitations of this study.

## 5. Conclusions

Within the limitations of this study, the following conclusions were obtained:

✓ There were significant increases in demineralizations adjacent to the bracket after placement in the cariogenic environment.
After placement in the cariogenic environment, the gingival and proximal enamel demineralization values and the amount of increase in demineralization values measured in the control group were found to be significantly lower than in the study groups. Thus, it was concluded that the area around the bracket creates a potential area for microbial plaque retention, leading to the development of demineralization in the cariogenic environment.
The fact that Victory metal and APC Clarity Advanced ceramic brackets exhibit less microbial plaque retention than others and cause less demineralization showed that they can be preferred in patients with poor oral hygiene.

## Figures and Tables

**Table 1:** Demineralization values of the groups on the gingival enamel surface adjacent to the bracket at T0 and T1 periods.

Gingival		Victory Metal ^1^	Gemini Metal ^2^	Clarity Self-Ligating ^3^	APC Clarity Advanced ^4^	Clarity Advanced ^5^	Control ^6^	*p*
T0	Mean ± SD	2.2 ± 0.8	2.9 ± 0.6	3.3 ± 0.8	3.1 ± 1.0	3.3 ± 0.9	2.4 ± 0.5	NS
T1	Mean ± SD	10.3 ± 1.8 ^2,3,4,5^	11.9 ± 1.8 ^3,5^	13.9 ± 2.7	11.9 ± 2.8 ^3^^,^⁵	16.7 ± 8.1	8.0 ± 0.8 ^1,2,3,4,5^	0.000 ^K^
T0/T1 difference	Mean ± SD	8.1 ± 2.2 ⁵	9.1 ± 2.1 ⁵	10.5 ± 2.7 ⁵	8.8 ± 2.8 ⁵	13.4 ± 7.8	5.6 ± 0.7 ^1,2,3,4,5^	0.000 ^K^
Intragroup difference *p*		0.001 ^W^	0.001 ^W^	0.001 ^W^	0.001 ^W^	0.001 ^W^	0.000 ^W^	

^K^ Kruskal–Wallis (Mann–Whitney U test); ^W^ Wilcoxon test; ^1^ Difference with Victory metal group *p* < 0.05; ^2^ Difference with Gemini metal group *p* < 0.05; ^3^ Difference with Clarity self-ligating group *p* < 0.05; ^4^ Difference with APC Clarity Advanced group *p* < 0.05; ^5^ Difference with Clarity Advanced group *p* < 0.05; ^6^ Difference with control group *p* < 0.05. T0 Before placing in the cariogenic environment; T1 28 days after placement in cariogenic environment; SD: Standard deviation; NS: Not significant.

**Table 2:** Demineralization values of the groups on the proximal enamel surface adjacent to the bracket at T0 and T1 periods.

Proximal		Victory Metal ^1^	Gemini Metal ^2^	Clarity Self-Ligating ^3^	APC Clarity Advanced ^4^	Clarity Advanced ^5^	Control ^6^	*p*
T0	Mean ± SD	2.8 ± 0.7	4.0 ± 1.3	4.0 ± 1.1	4.8 ± 1.5	4.3 ± 0.7	3.6 ± 0.7	NS
T1	Mean ± SD	14.3 ± 3.0 ^3,5^	15.4 ± 3.5	15.5 ± 1.6	14.3 ± 3.1 ^3,5^	18.6 ± 5.5	11.8 ± 1.1 ^1,2,3,4,5^	0.000 ^K^
T0/T1 difference	Mean ± SD	11.5 ± 3.1 ^5^	11.4 ± 4.4 ^5^	11.5 ± 2.0 ^5^	9.5 ± 2.7 ^1,2,3,5^	14.3 ± 5.6	8.2 ± 1.4 ^1,2,3,4,5^	0.000 ^K^
Intragroup difference *p*		0.001 ^W^	0.001 ^W^	0.001 ^W^	0.001 ^W^	0.001 ^W^	0.001 ^W^	

^K^ Kruskal–Wallis (Mann–Whitney U test); ^W^ Wilcoxon test; ^1^ Difference with Victory metal group *p* < 0.05; ^2^ Difference with Gemini metal group *p* < 0.05; ^3^ Difference with Clarity self-ligating group *p* < 0.05; ^4^ Difference with APC Clarity Advanced group *p* < 0.05; ^5^ Difference with Clarity Advanced group *p* < 0.05; ^6^ Difference with control group *p* < 0.05; T0 Before placing in the cariogenic environment; T1 28 days after placement in cariogenic environment; SD: Standard deviation; NS: Not significant.

**Table 3:** Demineralization values of the groups on the occlusal enamel surface adjacent to the bracket at T0 and T1 periods.

Occlusal		Victory Metal ^1^	Gemini Metal ^2^	Clarity Self-Ligating ^3^	APC Clarity Advanced ^4^	Clarity Advanced ^5^	Control ^6^	*p*
T0	Mean ± SD	1.7 ± 0.5	2.3 ± 1.0	2.5 ± 0.6	2.6 ± 0.8	2.3 ± 0.6	2.3 ± 0.5	NS
T1	Mean ± SD	8.4 ± 1.1 ^2,3,5^	9.0 ± 1.2	10.9 ± 5.3	8.4 ± 0.7 ^2,3,5^	9.6 ± 0.7	7.9 ± 0.7 ^2,3,5^	0.000 ^K^
T0/T1 difference	Mean ± SD	6.7 ± 1.1	6.7 ± 1.7	8.3 ± 5.5	5.8 ± 1.3 ^1,2,3,5^	7.3 ± 0.8	5.6 ± 0.7 ^1,2,3,5^	0.000 ^K^
Intragroup difference *p*		0.000 ^W^	0.001 ^W^	0.001 ^W^	0.001 ^W^	0.001 ^W^	0.000 ^W^	

^K^ Kruskal–Wallis (Mann–Whitney U test); ^W^ Wilcoxon test; ^1^ Difference with Victory metal group *p* < 0.05; ^2^ Difference with Gemini metal group *p* < 0.05; ^3^ Difference with Clarity self-ligating group *p* < 0.05; ^4^ Difference with APC Clarity Advanced group *p* < 0.05; ^5^ Difference with Clarity Advanced group *p* < 0.05; ^6^ Difference with control group *p* < 0.05; T0 Before placing in the cariogenic environment; T1 28 days after placement in cariogenic environment; SD: Standard deviation; NS: Not significant.

**Table 4:** Demineralization values of the groups on the gingival, proximal and occlusal enamel surface adjacent to the bracket at T0 and T1 periods.

			Victory Metal ^1^	Gemini Metal ^2^	Clarity Self-Ligating ^3^	APC Clarity Advanced ^4^	Clarity Advanced ^5^	Control ^6^	*p*
Gingival	T0	Mean ± SD	2.2 ± 0.8	2.9 ± 0.6	3.3 ± 0.8	3.1 ± 1.0	3.3 ± 0.9	2.4 ± 0.5	NS
	T1	Mean ± SD	10.3 ± 1.8 ^2,3,4,5^	11.9 ± 1.8 ^3,5^	13.9 ± 2.7	11.9 ± 2.8 ^3,5^	16.7 ± 8.1	8.0 ± 0.8 ^1,2,3,4,5^	0.000
	T0/T1 difference	Mean ± SD	8.1 ± 2.2 ^3,5^	9.1 ± 2.1 ⁵	10.5 ± 2.7 ⁵	8.8 ± 2.8 ⁵	13.4 ± 7.8	5.6 ± 0.7 ^1,2,3,4,5^	0.000
Proximal	T0	Mean ± SD	2.8 ± 0.7	4.0 ± 1.3	4.0 ± 1.1	4.8 ± 1.5	4.3 ± 0.7	3.6 ± 0.7	NS
	T1	Mean ± SD	14.3 ± 3.0 ^3,5^	15.4 ± 3.5	15.5 ± 1.6	14.3 ± 3.1 ^3,5^	18.6 ± 5.5	11.8 ± 1.1 ^1,2,3,4,5^	0.000
	T0/T1 difference	Mean ± SD	11.5 ± 3.1 ⁵	11.4 ± 4.4 ⁵	11.5 ± 2.0 ⁵	9.5 ± 2.7 ^1,2,3,5^	14.3 ± 5.6	8.2 ± 1.4 ^1,2,3,4,5^	0.000
Occlusal	T0	Mean ± SD	1.7 ± 0.5	2.3 ± 1.0	2.5 ± 0.6	2.6 ± 0.8	2.3 ± 0.6	2.3 ± 0.5	NS
	T1	Mean ± SD	8.4 ± 1.1 ^2,3,5^	9.0 ± 1.2	10.9 ± 5.3	8.4 ± 0.7 ^2,3,5^	9.6 ± 0.7	7.9 ± 0.7 ^2,3,5^	0.000
	T0/T1 difference	Mean ± SD	6.7 ± 1.1	6.7 ± 1.7	8.3 ± 5.5	5.8 ± 1.3 ^1,2,3,5^	7.3 ± 0.8	5.6 ± 0.7 ^1,2,3,5^	0.000

^1^ Difference with Victory metal group *p* < 0.05; ^2^ Difference with Gemini metal group *p* < 0.05; ^3^ Difference with Clarity self-ligating group *p* < 0.05; ^4^ Difference with APC Clarity Advanced group *p* < 0.05; ^5^ Difference with Clarity Advanced group *p* < 0.05; ^6^ Difference with control group *p* < 0.05; T0 before placing in the cariogenic environment; T1 28 days after placement in cariogenic environment; SD: Standard deviation; NS: Not significant.

## Data Availability

All data supporting the results of this study are included within the article.
